# Enhanced encapsulation and membrane retention of *Teleogryllus mitratus* protein via hydrophobic ion-pairing in nanostructured lipid carriers

**DOI:** 10.1016/j.ijpx.2026.100550

**Published:** 2026-04-24

**Authors:** Jirasit Inthorn, Suvimol Somwongin, Saranya Juntrapirom, Watchara Kanjanakawinkul, Andrea Heinz, Anette Müllertz, Thomas Rades, Wantida Chaiyana

**Affiliations:** a Department of Pharmaceutical Sciences, Faculty of Pharmacy, Chiang Mai University, Chiang Mai 50200, Thailand; b Chulabhorn Royal Pharmaceutical Manufacturing Facilities by Chulabhorn Royal Academy, Chon Buri 20180, Thailand; c Department of Pharmacy, LEO Foundation Center for Cutaneous Drug Delivery, University of Copenhagen, 2100 Copenhagen, Denmark; d Department of Pharmacy, Faculty of Health and Medical Sciences, University of Copenhagen, Universitetsparken 2, 2100 Copenhagen, Denmark; e Bioneer: FARMA, Department of Pharmacy, University of Copenhagen, Universitetsparken 4, Copenhagen 2100, Denmark; f Center of Excellence in Pharmaceutical Nanotechnology, Faculty of Pharmacy, Chiang Mai University, Chiang Mai 50200, Thailand; g Research Center of Deep Technology in Beekeeping and Bee Products for Sustainable Development Goals (SMART BEE SDGs), Chiang Mai University, Chiang Mai 50200, Thailand; h Multidisciplinary and Interdisciplinary School, Chiang Mai University, Chiang Mai 50200, Thailand

**Keywords:** Cricket, Protein hydrolysate, Hydrophobic ion pair, Nanocarrier, Nanodelivery system, Nanotechnology

## Abstract

This study aimed to enhance the topical delivery of *Teleogryllus mitratus* protein hydrolysate (TM) by forming hydrophobic ion-pair (HIP) complexes with dioctyl sodium sulfosuccinate (DS) and incorporating them into lipid-based nanocarriers. TM was extracted via an enzyme-assisted method and complexed with DS to form TM-HIP. Chitosan nanoparticles (CNP), nanoemulsions (NE), and nanostructured lipid carriers (NLC) containing TM or TM-DS were prepared and characterized for particle size, polydispersity, zeta potential, encapsulation efficiency (EE), loading capacity (LC), and in vitro release. Membrane retention was assessed using Strat-M® membranes in Franz diffusion cells. The results showed that TM successfully formed a HIP complex with DS, resulting in an increased diffusion coefficient. All nanocarriers exhibited nanoscale particle sizes (∼70–300 nm), narrow distributions (PDI 0.17–0.26), and stable zeta potentials (−30 to −37 mV). Lipid-based nanocarriers containing TM-DS demonstrated the highest EE (TM-DS-NE: 76.8 ± 0.5%; TM-DS-NLC: 81.9 ± 2.4%) and sustained release, while CNP showed lower EE (17.6 ± 3.1%). Membrane retention studies revealed that TM-DS-NE (49.9 ± 0.7 μg/cm^2^) and TM-DS-NLC (50.1 ± 3.1 μg/cm^2^) achieved significantly higher protein deposition than TM-CNP (1.6 ± 0.8 μg/cm^2^), TM-NE (6.4 ± 1.2 μg/cm^2^), TM-NLC (9.2 ± 1.7 μg/cm^2^), or TM solution (1.2 ± 0.7 μg/cm^2^), with NLC identified as the most effective carrier. Therefore, it can be concluded that hydrophobic ion-pairing of TM with DS enhanced compatibility with lipid-based nanocarriers, resulting in improved encapsulation and membrane retention. The combination of protein lipophilicity, carrier composition, and nanoscale size effectively promoted delivery into the Strat-M® membranes. Further clinical studies are recommended to validate efficacy and safety under physiologically relevant conditions.

## Highlights

• *T. mitratus* protein hydrolysate (TM) was a bioactive protein for topical use.
• Hydrophobic ion-pairing (HIP) with sodium docusate (DS) enhanced TM lipophilicity.
• TM–DS complexes enabled efficient incorporation into lipid-based nanocarriers.
• Nanoemulsions and nanostructured lipid carriers (NLC) were achieved.
• TM–DS–NLCs enabled improved protein retention in Strat-M® membranes.

## Graphical abstract

**Figure:**
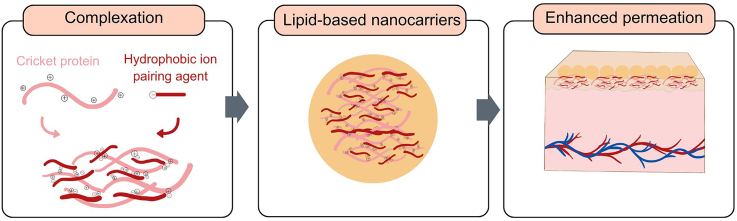

## 1 Introduction

The increasing global demand for sustainable and nutrient-dense protein sources has driven considerable interest in edible insects, which offer a low environmental footprint and high nutritional value compared with conventional livestock (Nowakowski et al., 2022). Insects can be produced with less land-, water-, and feed-use, and generate lower greenhouse gas emissions, making them a highly attractive option in the context of food security and climate change mitigation (Van Huis and Oonincx, 2017; Halloran et al., 2016). Among edible insects, crickets have emerged as particularly promising candidates due to their high protein content, favorable amino acid composition, and efficient farming requirements. In Thailand, cricket farming has been actively promoted by the Department of Livestock Development as an alternative protein source for both food and feed, reflecting their growing commercial and nutritional relevance (Ordonez-Araque and Egas-Montenegro, 2021; Krongdang et al., 2023). Species such as *Gryllus bimaculatus*, *Acheta domesticus*, and *Teleogryllus mitratus* exhibit strong potential for large-scale production. In particular, *T. mitratus* is notable for its high protein yield, broad availability, and applicability in generating bioactive compounds, positioning it also as a compelling source for cosmeceutical and therapeutic development (Nowakowski et al., 2022; Magara et al., 2021).

Cricket proteins constitute a complex nutritional matrix, comprising essential amino acids, bioactive peptides, minerals, lipids, vitamins, and chitosan fibers, thereby representing a versatile bioresource for applications in food science and cosmeceutical formulations (Nowakowski et al., 2022; Magara et al., 2021). Bioactive proteins and peptides derived from crickets have been reported to exhibit antioxidant, anti-inflammatory, and anti-aging activities, highlighting their potential for cosmeceutical applications (Messina et al., 2019; Hierro et al., 2020; Yeerong et al., 2021). Enzymatic hydrolysis of cricket proteins further enhances their solubility, generates smaller bioactive peptides, and increases functional efficacy *in vitro* (Hall et al., 2017; Hall et al., 2018). Despite these advantages, the topical delivery of *T. mitratus* proteins remains challenging, as hydrophilic macromolecules are inherently limited in their ability to penetrate the stratum corneum and reach deeper, viable skin layers (Mortazavi and Moghimi, 2022). Consequently, achieving effective skin deposition of cricket-derived proteins is a major barrier to their cosmeceutical utilization.

Nanotechnology offers a transformative approach to overcome the delivery challenges of bioactive proteins. Nanocarriers, including polymeric nanoparticles and lipid-based systems, can protect proteins from degradation, enhance solubility, enable controlled release, and facilitate transport across biological barriers (Kaul et al., 2018; Gupta et al., 2022; Sharma et al., 2023). Lipid-based nanocarriers, such as nanoemulsions and nanostructured lipid carriers (NLC), are particularly suitable for dermal delivery due to their biocompatibility, occlusive properties, and ability to interact with the lipid components of the skin (Souto et al., 2022; Montenegro et al., 2016). These systems can improve retention of bioactive compounds within the upper skin layers while limiting systemic absorption, making them attractive for cosmeceutical and dermatological applications (Gupta et al., 2022; Ganesan and Choi, 2016). However, the incorporation of hydrophilic proteins into lipid matrices is often inefficient, leading to low entrapment efficiency and suboptimal delivery. This limitation reduces the therapeutic or cosmetic performance of protein-based formulations and necessitates strategies to enhance protein–lipid compatibility (Wang et al., 2015).

Hydrophobic ion-pairing (HIP) has emerged as a promising method to address this challenge. HIP involves the formation of stable complexes between positively charged protein residues and anionic surfactants, resulting in proteins with increased lipophilicity (Ristroph and Prud'homme, 2019; Xin et al., 2023). This transformation enhances the compatibility of hydrophilic proteins with lipid-based carriers, facilitating higher encapsulation efficiency, improved stability, and controlled release (Ristroph and Prud'homme, 2019; Xin et al., 2023). HIP can modulate the surface characteristics of proteins, promoting their interaction with lipid droplets in nanoemulsion and NLC formulations, ultimately improving deposition within skin-mimicking membranes (Ristroph and Prud'homme, 2019). By increasing protein lipophilicity while preserving functional activity, HIP provides a rational strategy to overcome the solubility and permeability limitations of macromolecular proteins in dermal applications. Despite the recognized sustainability, nutritional value, and bioactive potential of *T. mitratus* proteins (Nischalke et al., 2020), their application in topical formulations is currently limited. Therefore, this study aimed to develop TM-HIP complexes and incorporate them into various nanocarrier systems to increase entrapment efficiency and enhance penetration. The optimized nanocarrier systems are expected to provide a foundation for the future development of cricket protein–based cosmeceutical formulations and to introduce a novel strategy for improving the dermal delivery of cricket-derived bioactive compounds.

## 2 Materials and methods

### 2.1 *T. mitratus* cricket materials

*T. mitratus* in frozen form was obtained from a local farm in Chiang Mai, Thailand, and freeze-dried for 24 h to remove moisture. Following this, the dried *T. mitratus* were subjected to cold-press extraction using an FEA-100SS-M-H-TC screw press (Energy Friend Ltd., Chiang Mai, Thailand) to remove oil (Inthorn et al., 2024). The remaining *T. mitratus* residue was ground into powder using a Grinder PG500 (Spring Green Evolution Co. Ltd., Bangkok, Thailand) and subsequently treated with absolute ethanol to remove pigments, remaining fat and low-molecular-weight impurities, following the procedure described by Inthorn et al. (2024). The resulting powder was stored in sealed containers until further experiments.

### 2.2 Chemical materials

The bicinchoninic acid (BCA) protein assay kit was purchased from EMD Millipore Corp. (Darmstadt, Germany). Subtilisin A (protease from *Bacillus licheniformis*, 2.4 Anson units/g, E.C. 3.4.21.62), sodium docusate or dioctyl sodium sulfosuccinate (DS), sodium deoxycholate (SC), and Tween® 85 were obtained from Sigma-Aldrich (Darmstadt, Germany). 6-Hydroxy-2,5,7,8-tetramethyl chroman-2-carboxylic acid (Trolox) and 2,2-diphenyl-1-picrylhydrazyl (DPPH) were purchased from Merck KGaA (Darmstadt, Germany). Sodium oleate (SO) was supplied by Glentham Life Science (Corsham, UK). Sorbitol and shea butter, both cosmetic grades, were purchased from Namsiang Co., Ltd. (Bangkok, Thailand). Absolute ethanol, methanol, *n*-butanol, hydrochloric acid (37%), sodium hydroxide (NaOH), hydrochloric acid (HCl), trisodium citrate, and glacial acetic acid, all of analytical grade, were supplied by RCI Labscan Ltd. (Bangkok, Thailand). Phosphotungstic acid was purchased from Loba Chemie (Mumbai, India). Strat-M® membrane was purchased from Merck Millipore Ltd. (County Cork, Ireland). The FastCast™ acrylamide kit, Laemmli buffer, Coomassie brilliant blue G-250, and Precision Plus Protein™ Dual Xtra prestained protein standards (Bio-Rad Laboratories Inc., No. 1610377), a combination of standard proteins with relative molecular masses ranging from 2 to 250 kDa, were purchased from Bio-Rad Laboratories Inc. (Hercules, CA, USA).

### 2.3 Enzyme-assisted preparation of *T. mitratus* cricket protein extract

*T. mitratus* protein hydrolysate (TM) was extracted using an enzyme-assisted method following the procedure described by Hall et al. (2018). Briefly, *T. mitratus* powder was dispersed in deionized (DI) water, pasteurized at 90 °C for 15 min, cooled to room temperature, adjusted to pH 8.0, and then treated with protease (subtilisin A) at an enzyme-to-substrate ratio of 1:10 *w/w*. Enzymatic extraction was carried out for 4 h at 60 °C under moderate stirring using a hot plate stirrer (IKA® C-MAG HS7, IKA Werke GmbH & Co. KG, Staufen, Germany). Following extraction, the mixture was pasteurized to inactivate the enzyme and cooled to room temperature. The extract was centrifuged at 3200 ×*g* for 20 min at 4 °C using an ultracentrifuge (MPW-352R, MPW Med. Instruments, Warsaw, Poland). The supernatant was collected, the pH adjusted to 7.0 and then lyophilized using a CHRIST Beta 2–8 LDplus freeze dryer (Martin Christ Gefriertrocknungsanlagen GmbH, Osterode am Harz, Germany). All extraction steps were performed under light-protected conditions. The resulting dried TM was stored in sealed aluminum foil bags at −20 °C until further use.

### 2.4 Determination of total protein content of cricket extract by bicinchoninic acid (BCA) assay

The total protein concentration of the TM obtained above was assessed via a BCA assay following the methodology outlined by Mba et al. (2021). In brief, 25 μl of TM solution was combined with 200 μl of BCA working reagent and incubated at 37 °C for 30 min. The absorbance was subsequently evaluated at 562 nm using a microplate reader (CLARIOstar PLUS, BMG Labtech, Ortenberg, Germany). The absorbance of each sample was used to calculate the total protein content, expressed as g bovine serum albumin (BSA)/g sample, using the equation obtained from the standard curve (y = 0.0023x + 0.0472, R^2^ = 0.9965), where y represents absorbance and x represents BSA concentration. A calibration curve (Fig. S1) was constructed using BSA (0–250 μg/ml). The limit of detection (LOD) and limit of quantification (LOQ) were calculated from the standard deviation of the y-intercept (Sb) and the slope (m) of the calibration curve using LOD = (3.3 × Sb)/m and LOQ = (10 × Sb)/m, yielding values of 5.9 and 17.7 μg/ml, respectively. Three replicates of each experiment were conducted.

### 2.5 Protein profiling of cricket extracts by sodium dodecyl sulfate-polyacrylamide gel electrophoresis (SDS-PAGE)

The protein profile of TM was analyzed using the SDS–PAGE technique (Fashakin et al., 2023). The aqueous solution of TM, at a concentration of 10 mg/ml, was mixed with Laemmli sample buffer at a ratio of 3:1. The resulting mixture was denatured at 70 °C for 10 min and then loaded onto a 12% polyacrylamide gel (FastCast™ Acrylamide Kit), along with a standard protein marker. Electrophoresis was performed using a vertical polyacrylamide gel electrophoresis system (Mini-PROTEAN Tetra Cell, Bio-Rad Laboratories Inc., CA, USA) at 100 *V* for approximately 45 min. Following electrophoresis, the protein bands were fixed in a solution containing 40% methanol and 10% acetic acid for 30 min. The gel was then washed, stained with Coomassie Brilliant Blue G-250 for 1 h, and rinsed with 10% acetic acid, followed by a final wash with DI water. Finally, the gel was imaged using a Gel Doc imaging system (Bio-Rad Laboratories Inc., CA, USA).

### 2.6 Chemical modification–based preparation of *T. mitratus* cricket chitosan

*T. mitratus* cricket chitosan was prepared using a chemical modification procedure following the method described by Inthorn et al. (2024). Initially, 50 g of *T. mitratus* powder was subjected to deproteinization by treatment with 500 ml of 1.0 M NaOH at 80 °C for 6 h under moderate stirring using a hot plate stirrer (IKA® C-MAG HS7, IKA Werke GmbH & Co. KG, Staufen, Germany). The resulting deproteinized chitin was reco*v*ered by filtration and thoroughly washed until the filtrate reached neutral pH. For demineralization, the deproteinized chitin pellets were treated with 500 ml of 0.25 M HCl at 85 °C for 15 min. The demineralized chitin was then washed repeatedly with DI water to reach neutral pH and allowed to dry. Deacetylation was performed to convert chitin into chitosan by immersing the demineralized chitin in 67% *w*/*v* NaOH at a powder-to-solvent ratio of 1:20. The mixture was maintained at 110 °C for 2 h under moderate stirring using a hot plate stirrer (IKA® C-MAG HS7, IKA Werke GmbH & Co. KG, Staufen, Germany). The resulting chitosan pellets were filtered, washed until neutral pH, and dried overnight at 60 °C in a hot-air oven (UM500, Memmert, Schwabach, Germany). The resulting cricket chitosan was stored in sealed aluminum foil bags until further use.

### 2.7 Preparation of *T. mitratus* cricket protein–hydrophobic ion-pair complexes (TM-HIP)

TM was complexed with various HIP agents, including DS, SC, and SO. The effect of pH on protein precipitation was first evaluated to optimize the experimental conditions. An HCl solution with the lowest pH that produced a non-turbid solution was selected for subsequent TM-HIP formation. Briefly, each HIP agent was dissolved at a concentration of 15 mg/ml in HCl solutions with pH values ranging from 2.0 to 4.0. TM was also prepared at the same concentration over the same pH range. The solutions of either TM or HIP agents were then mixed and stirred at 400 rpm for 2 h using a hot plate stirrer (IKA® C-MAG HS7, IKA Werke GmbH & Co. KG, Staufen, Germany). Turbidity was measured at 600 nm using a SpectroStar Nano plate spectrophotometer (CLARIOstar®, BMG LABTECH, Ortenberg, Germany) to identify the lowest pH at which the HIP agents did not precipitate. The optimal pH for each HIP agent was subsequently used to determine the optimal TM-to-HIP ratio. Briefly, TM (5 mg/ml) was mixed with varying concentrations of each HIP agent under stirring at 400 rpm for 2 h using a hot plate stirrer (IKA® C-MAG HS7, IKA Werke GmbH & Co. KG, Staufen, Germany). The resulting TM-HIP suspensions were measured for turbidity at 600 nm and zeta potential using a Zetasizer. The mixtures were centrifuged at 10,000 ×*g* for 20 min at 25 °C using a laboratory centrifuge (MPW-352R, MPW Med. Instruments, Warsaw, Poland). The free protein in the supernatant was quantified using a BCA protein assay kit (EMD Millipore Corp., Darmstadt, Germany), as described in Section 2.4. The precipitation efficiency of the TM-HIP complexes was calculated using the following equation: Precipitation efficiency (%) = 100 − [(A/B) × 100], where A is the amount of free protein in the supernatant and B is the total protein content (Griesser et al., 2017).

### 2.8 Characterization of TM-HIP

The TM-HIP complex selected based on the highest precipitation efficiency was used for further characterization. Prior to characterization, the selected TM-HIP complex was scaled up. The TM solution was prepared at a concentration of 0.5% *w/w*, and the HIP agent solution was prepared at a concentration of 0.25% *w/w*. The TM solution was mixed with an equal volume of the HIP agent solution under stirring at 400 rpm for 2 h. Regarding the scale-up process, the resulting TM-HIP suspension was directly subjected to lyophilization using a freeze dryer (CHRIST Beta 2–8 LDplus, Martin Christ Gefriertrocknungsanlagen GmbH, Osterode am Harz, Germany) without centrifugation due to the minimal amount of precipitate formed in the preliminary study. The resulting dried TM-HIP was stored in sealed aluminum foil bags until further characterization by Fourier-transform infrared (FTIR) spectroscopy and X-ray diffraction (XRD).

#### 2.8.1 FTIR spectroscopy

The functional groups of TM, selected HIP agent, their physical mixture, and selected TM-HIP complex were analyzed in attenuated total reflection (ATR) mode using FTIR spectroscopy (ALPHA II, Bruker, Karlsruhe, Germany). Briefly, each sample with a thickness of approximately 1 mm was placed directly on the diamond crystal plate of the instrument, and the pressure arm was applied. FTIR spectra were recorded over a wavenumber range of 4000–400 cm^−1^ and plotted as transmittance versus wavenumber (cm^−1^) (Ibitoye et al., 2018).

#### 2.8.2 XRD

The XRD patterns of TM, selected HIP agent, their physical mixture, and selected TM-HIP complex, were recorded using an X-ray diffractometer (D2 PHASER, Bruker, Karlsruhe, Germany) operated at 30 kV and 10 mA. Samples were loaded into specimen rings (25 mm) and scanned over a 2*θ* range of 5–80° with a step size of 0.2°/s. The XRD diffractograms are presented as intensity (arbitrary units) versus the 2*θ* angle (degrees) (Sutar et al., 2021).

#### 2.8.3 Determination of distribution coefficient (log D)

The log D of TM and the TM-DS was determined following the method of Claus et al. (2023) with some modifications. Briefly, TM (20 mg) or TM-DS (30 mg, equivalent to 20 mg TM) was dissolved in 5 ml of *n*-butanol and sonicated for 30 min using an ultrasonicator (Elmasonic S30H, Elma Schmidbauer GmbH, Singen, Germany). Subsequently, 5 ml of DI water was added, and the resulting mixture was transferred to a 50 ml centrifuge tube and shaken horizontally in a shaking incubator (WNB10, Memmert GmbH & Co. KG, Schwabach, Germany) set at 500 rpm for 2 h at room temperature. The samples were then centrifuged at 12,000 ×*g* for 10 min to separate the *n*-butanol and aqueous phases. Aliquots (500 μl) of each phase were collected and dried at room temperature in a fume hood (EFD-4B8 Esco Frontier™ Duo Fume Hood, Esco Lifesciences (Thailand) Co., Ltd., Bangkok, Thailand) to remove residual *n*-butanol. The dried samples were redissolved in DI water, sonicated for 30 min, and shaken at 500 rpm for 8 h in a shaking incubator (SV14/22, Memmert GmbH & Co. KG, Schwabach, Germany) prior to protein quantification using the BCA assay as described in section 2.4. Log D was calculated using the equation: log D_*n*-butanol/water_ = log (C_*n*-butanol_/C_water_), where C_*n*-butanol_ and C_water_ refer to the concentrations of protein in the *n*-butanol and DI water phases, respectively. Three replicates of each experiment were conducted.

### 2.9 Determination of antioxidant activity of TM and TM-DS

The antioxidant activity of the extracts was evaluated using the DPPH assay following a previously described method of Chaiyana et al. (2024). The effect of the HIP complexation process on antioxidant activity was also investigated by comparing the protein hydrolysate before complex formation (TM) with that recovered following HIP complexation (TM-DS) and subsequent dissociation. TM-DS was dispersed in absolute ethanol, subsequently diluted with DI water, and stirred at 200 rpm for 6 h using a hot plate stirrer (IKA® C-MAG HS7, IKA Werke GmbH & Co. KG, Staufen, Germany) to achieve complete dissociation. In addition, the effect of acidic conditions (pH 2.0) used during the complexation process was evaluated by dissolving the TM protein hydrolysate in 17.5 mM HCl solution (pH 2.0) and subsequently assessing its antioxidant acti*v*ity. In brief, 20 μl of various concentrations of the sample solution was mixed with 180 μl of 167 μM DPPH^•^ methanolic solution and incubated in the dark at ambient temperature for 30 min. The absorbance of the resulting mixture was measured at 520 nm using a microplate reader (CLARIOstar®, BMG LABTECH, Ortenberg, Germany). The percentage inhibition against DPPH^•^ radicals was calculated using the following equation: DPPH^•^ radical inhibition (%) = [(A–B)/A] × 100, where A is the absorbance of the control (without sample) and B is the absorbance of the sample after blank correction. The half-maximal inhibitory concentration (IC_50_) values were calculated using GraphPad Prism version 10.6.1 (GraphPad Software Inc., La Jolla, CA, USA). Three replicates of each experiment were conducted.

### 2.10 Development of nanocarriers containing TM

#### 2.10.1 Chitosan nanoparticles with TM (TM-CNP)

TM-CNP were prepared using the ionotropic gelation method, following the procedure described by Inthorn et al. (2024), with some modifications. The compositions of TM-CNP are presented in Table 1. Briefly, 1.0 g of cricket chitosan, 0.4 g of TM, and 0.4 g of polysorbate-80 were dissolved in 300 ml of acetic acid solution (0.4% *v*/v) and homogenized using a high-speed homogenizer (T25 ULTRA-TURRAX® digital, IKA Werke GmbH & Co. KG, Staufen, Germany) at 3000 rpm for 5 min. Following this, 100 ml of a 1% *w/w* trisodium citrate solution was added dropwise to the cricket chitosan solution at a flow rate of 1.0 ml/min while maintaining continuous homogenization at 3000 rpm. The final concentrations of TM, cricket chitosan, and trisodium citrate were 0.10, 0.25, and 0.25% *w/w*, respectively. The pH of the TM-CNP suspension was adjusted to 4.5 using glacial acetic acid.

**Table 1:** The compositions of nanocarriers containing *T. mitratus* cricket protein hydrolysate and its complex.

Ingredients	Concentration (% *w/w*)						
	TM-SOL	TM-CNP	TM-NE	TM-NLC	TM-CNP -NE	TM-DS -NE	TM-DS -NLC
TM	0.1	0.1	0.1	0.1	–	–	–
Dried TM-CNP	–	–	–	–	0.7	–	–
TM-DS	–	–	–	–	–	0.15	0.15
TM chitosan	–	0.25	–	–	–	–	–
TM oil	–	–	5	4	5	5	4
Shea butter	–	–	–	1	–	–	1
Tween® 80	–	0.1	–	–	–	–	–
Tween® 85	–	–	5	5	5	5	5
Trisodium citrate	–	0.25	–	–	–	–	–
Sorbitol	–	–	5	5	5	5	5
DI water q.s.	100	100	100	100	100	100	100

Note: TM = *T. mitratus* cricket protein hydrolysate; TM-DS = the complex of *T. mitratus* cricket protein hydrolysate and dioctyl sodium sulfosuccinate; TM-SOL = *T. mitratus* cricket protein hydrolysate aqueous solution; TM-CNP = chitosan nanoparticles with *T. mitratus* cricket protein hydrolysate; TM-NE = nanoemulsion containing the *T. mitratus* cricket protein hydrolysate; TM-NLC = nanostructured lipid carriers containing the *T. mitratus* cricket protein hydrolysate; TM-CNP-NE = nanoemulsion containing chitosan nanoparticles with *T. mitratus* cricket protein hydrolysate; TM-DS-NE = nanoemulsion containing the complex of *T. mitratus* cricket protein hydrolysate and dioctyl sodium sulfosuccinate; TM-DS-NLC = nanostructured lipid carriers containing the complex of *T. mitratus* cricket protein hydrolysate and dioctyl sodium sulfosuccinate.

#### 2.10.2 Oil-in-water (O/W) nanoemulsion

The O/W nanoemulsions with TM from Section 2.3 (TM-NE), TM-CNP from Section 2.10.1 (TM-CNP-NE), and TM-DS from Section 2.7 (TM-DS-NE) were developed. TM, TM-CNP, or TM-DS were incorporated into the oil phase at a concentration corresponding to 0.1% *w/w* TM. In the case of TM-CNP and TM-DS, each suspension was first lyophilized using a CHRIST Beta 2–8 LDplus freeze dryer (Martin Christ Gefriertrocknungsanlagen GmbH, Osterode am Harz, Germany) at −50 °C for 72 h prior to incorporation. The compositions of each nanoemulsions (TM-NE, TM-CNP-NE, and TM-DS-NE) are presented in Table 1. In brief, O/W nanoemulsions were prepared using high-pressure homogenization, following the method described in our previous study (Chaiyana et al., 2024), with 5% *w/w T. mitratus* oil as the oil phase, 5% *w/w* Tween® 85 as the emulsifier, 5% *w/w* sorbitol as the humectant, and water added to 100% *w/w*. All components were mixed using a high-shear homogenizer (T25 ULTRA-TURRAX® digital, IKA Werke GmbH & Co. KG, Staufen, Germany) at 12,000 rpm for 5 min and then subjected to five cycles of high-pressure homogenization (APV 1000, Wilmington, MA, USA) at 300 bar.

#### 2.10.3 Nanostructured lipid carriers (NLC)

NLC containing TM (TM-NLC) and TM-DS (TM-DS-NLC) were prepared under the same processing conditions as the corresponding nanoemulsions, except that 1.0 g of cricket oil was replaced with 1.0 g of shea butter. The compositions of each NLC formulation are presented in Table 1. The dried TM or TM-DS was dispersed in the lipid phase of the NLC at a final concentration equivalent to 0.1% *w/w* TM.

### 2.11 Characterization of nanocarriers

#### 2.11.1 Physical appearance, particle size, polydispersity index (PDI), and zeta potentials

TM-CNP, TM-NE, TM-NLC, TM-CNP-NE, TM-DS-NE, and TM-DS-NLC formulations were evaluated for their visual characteristics, particle or droplet size, PDI, and zeta potential. The size and PDI were measured using dynamic light scattering with a Zetasizer (version 5.00, Malvern Instruments Ltd., Malvern, UK), while zeta potential was determined via electrophoretic light scattering using the same instrument. Prior to all measurements, each nanocarrier formulation was diluted tenfold with DI water to pre*v*ent excessive particle proximity. The same dilution was used for all formulations. Data are presented as mean ± standard deviation (SD), based on 10 repeated measurements per run, with three independent runs for each sample.

#### 2.11.2 Physical stability

The physical stability of the nanocarrier formulations was evaluated following a centrifugation method (Chaiyana et al., 2024). Each formulation was centrifuged using a MINI-10 K+ microcentrifuge (Hangzhou Miu Instruments Co., Ltd., Hangzhou, China) at 5000 rpm for 15 min. The external appearance, particularly regarding phase separation, was evaluated by visual inspection. Additionally, accelerated stability testing was conducted by subjecting all formulations to six temperature-cycling cycles, consisting of heating at 45 °C for 48 h followed by cooling at 4 °C for 48 h. Upon completion of the cycles, visual inspection was performed again to assess external appearance and phase separation. Particle size, PDI, and zeta potential were subsequently measured as described in Section 2.11.1.

#### 2.11.3 Morphology of nanocarriers

The shape and morphology of all nanocarriers were examined by transmission electron microscopy (TEM). Prior to TEM analysis, each nanocarrier formulation was diluted tenfold with DI water. A small volume of each diluted sample was placed onto a 300-mesh carbon-coated copper grid. Negative staining was performed using a 1% *w*/*v* phosphotungstic acid solution to enhance contrast, followed by vacuum drying for 30 min. TEM images were acquired at an accelerating voltage of 100 kV and magnifications of 20,000–30,000× using a JEOL JEM-2010 microscope (JEOL Ltd., Tokyo, Japan) (Jandang et al., 2024).

### 2.12 Entrapment efficiency (EE) and loading capacity (LC)

Each nanocarrier formulation was evaluated for its EE and LC using indirect analytical methods (Silva et al., 2022; Yeerong et al., 2024a). Briefly, a portion of each nanocarrier formulation was subjected to centrifugation using a centrifugal filter unit (Amicon Ultra-15, MWCO 100 kDa, Merck KGaA, Darmstadt, Germany) in an MPW-352R centrifuge (MPW Med. Instruments, Warsaw, Poland) at 10,000 rpm for 15 min. The amount of unencapsulated TM in the filtrate was quantified using the BCA assay, as described in Section 2.4, and subsequently used to calculate EE and LC. Both EE and LC were determined by comparing the amount of free protein to the total protein initially incorporated into the formulation, using the following equations: EE (% *w/w*) = [(W1–W2)/W1] × 100 and LC (% *w/w*) = [(W1–W2)/W3] × 100, where W1 represents the total protein content incorporated into the nanocarriers suspension, W2 represents the total protein content measured from the filtrate, and W3 represents mass of internal phase of nanocarrier. All experiments were performed in triplicate.

### 2.13 *In vitro* release study

The *in vitro* release of TM from nanocarrier formulations was evaluated in comparison with an aqueous TM solution using a dialysis bag method, as previously described (Gomez-Lazaro et al., 2024; Zhang et al., 2023). Prior to the experiment, dialysis tubing (MWCO 6–8 kDa; Spectra/Por®, Repligen Corp., Rancho Dominguez, CA, USA) was cut to appropriate lengths and soaked overnight in phosphate-buffered saline (PBS, pH 5.5). The saturated dialysis tubes were then gently blotted dry with tissue paper, and one end was sealed with coated aluminum wire. Subsequently, 2 ml of each formulation was introduced into the prepared dialysis tubes, and the opposite end was carefully secured with coated aluminum wire to prevent air bubbles. Each full dialysis tube was then placed in a beaker containing 20 ml of PBS (pH 5.5) and maintained on a magnetic stirrer (IKA® C-MAG HS7, IKA Werke GmbH & Co. KG, Staufen, Germany) at 100 rpm and 32 °C. At predetermined time intervals (0.5, 1, 2, 4, 6, 8, 24, and 48 h), 1 ml of the release medium was withdrawn and immediately replaced with an equal volume of fresh PBS (pH 5.5) to maintain sink conditions. The amount of TM released was quantified using the BCA assay as described in Section 2.4. All experiments were conducted in duplicate.

### 2.14 Strat-M® membrane permeation and retention study

The membrane permeation and retention of TM from each nanocarrier formulations were evaluated using vertical Franz diffusion cells (Velp Scientific Inc., Milano, Italy), as previously described (Arce et al., 2020; Ossowicz-Rupniewska et al., 2021). As protein was the major component of TM, the use of biological skin models could result in protein-related analytical interference. Therefore, in the present study, a protein-free Strat-M® membrane (Merck Millipore Ltd., County Cork, Ireland) was used as an artificial skin model. The Strat-M® membrane has a multilayer structure (∼300 μm thick), comprising a dense top layer supported by porous polyethersulfone layers and a polyolefin non-woven backing, designed to mimic the epidermis, dermis, and subcutaneous layers of human skin (Haq et al., 2018). The membrane was mounted between the donor and receptor compartments of the diffusion cell. The receptor compartment was filled with PBS (pH 7.4) and maintained at 32 °C, with continuous stirring at 400 rpm using a magnetic stirrer to ensure sink conditions. Subsequently, 1.0 g of each nanocarrier formulation was applied to the membrane surface in the donor compartment. Samples of the receptor medium were withdrawn at predetermined time points (0.5, 1, 2, 4, 6, 8, and 24 h), and replaced by an equal volume of fresh PBS (pH 7.4) immediately after sampling. The permeated TM content was quantified using the BCA assay. Following the 24-h permeation study, membrane retention was assessed by carefully removing the Strat-M® membranes, rinsing them with DI water, cutting them into small pieces, and sonicating them in PBS (pH 7.4). The resulting extracts were centrifuged at 3200 ×*g* for 20 min, and the supernatants were collected for protein quantification using the BCA assay, as described in Section 2.4. All experiments were performed in triplicate.

### 2.15 Statistical analysis

Data are presented as mean ± standard deviation (SD). Statistical analyses were performed using GraphPad Prism version 10.6.1 (GraphPad Software Inc., La Jolla, CA, USA). Differences among multiple groups were assessed by one-way analysis of variance (ANOVA) followed by Tukey's post hoc test, while comparisons between two groups were analyzed using a paired *t*-test. A *p*-value of <0.05 was considered statistically significant.

## 3 Results and discussion

### 3.1 Enzyme-assisted *T. mitratus* protein (TM) and *T. mitratus* cricket chitosan

The protein extraction yield from *T. mitratus* was 59.3 ± 1.3% on a dry-weight basis. The total protein content of the extract was 0.46 ± 0.01 g BSA equivalent/g extract, indicating that nearly half of the TM mass consisted of protein. The protein profile of TM, assessed by SDS–PAGE (Fig. S2), exhibited faint, diffuse bands predominantly in the low molecular weight region (<15 kDa). This pattern suggested that TM predominantly contained low-molecular-weight peptides rather than intact high-molecular-weight proteins. The absence of protein bands in the higher molecular weight regions is likely due to partial protein degradation or the formation of hydrolyzed protein fragments, consistent with the use of subtilisin A in the extraction process. Additionally, the smearing observed near the lower molecular weight range may reflect a heterogeneous mixture of peptides with varying sizes. The lower molecular weight fractions of protein hydrolysates from crickets (*Acheta domesticus*) have been reported to exhibit cosmetic potential, with smaller peptides showing stronger matrix metalloproteinase-1 (MMP-1) inhibitory activity and greater DPPH^•^ radical scavenging capacity (Yeerong et al., 2024b). This effect may be related to the potentially improved bioavailability of smaller peptides, which could contribute to more effective inhibition of MMP-1, a key enzyme in collagen degradation, and offer more antioxidant protection against free radical-induced skin aging (Yeerong et al., 2024b). Aside from the protein extract, chitosan was also successfully extracted from *T. mitratus*, yielding small yellowish-brown pellets with a yield of 4.4 ± 0.7% on a dry-weight basis, which is comparable to values reported previously by Inthorn et al. *(*2024*)**.*

### 3.2 *T. mitratus* cricket protein–hydrophobic ion-pair complexes (TM-HIP)

**Fig. 1:**
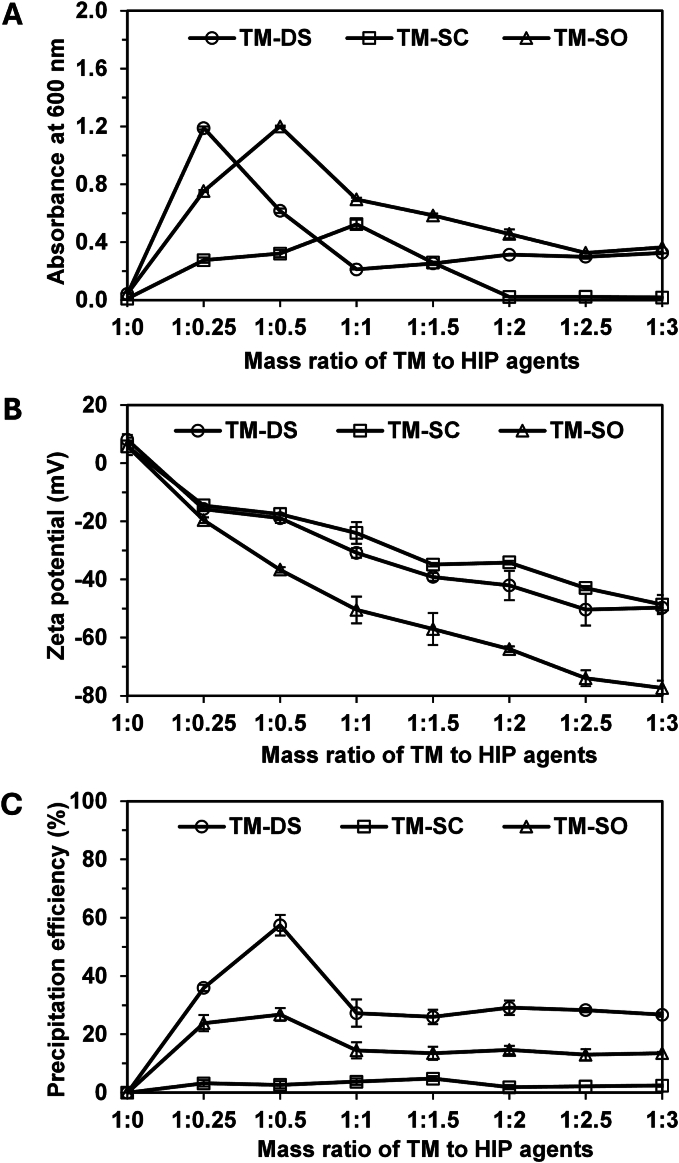
Turbidity (A), zeta potential (B), and precipitation efficiency (C) of *T. mitratus* cricket protein hydrolysate (TM) complexes prepared at different mass ratios using various hydrophobic ion-pairing (HIP) agents, including dioctyl sodium sulfosuccinate (TM-DS), sodium deoxycholate (TM-SC), and sodium oleate (TM-SO).

The formation of TM-HIP was strongly dependent on both pH and the TM-to-HIP agent ratio. TM and DS remained fully soluble at pH < 2.5, whereas SC and SO precipitated within the pH range of 2.0–2.5 (Fig. S3A), indicating that DS provided superior solubility under acidic conditions. Based on these observations, pH 2.0 was selected for TM-DS complex optimization, while pH 2.75 was used for TM-SC and TM-SO complexes. The effect of the TM-to-HIP agents ratio on complex formation was evaluated by varying the ratio from 1.0:0.0 to 1.0:3.0. The ratios corresponding to maximum precipitation differed among the systems (Fig. S4), highlighting that both pH and TM-to-HIP agents ratio critically determine TM-HIP formation. The most pronounced precipitation visually observed at the ratio of 1.0:0.25, 1.0:1.0, and 1.0:0.5 for TM-DS, TM-SC, and TM-SO, respectively. In addition to the visual observations, these differences were further confirmed by turbidity measurements (Fig. 1A), zeta potential analysis (Fig. 1B), and precipitation efficiency data (Fig. 1C). Turbidity measurements at 600 nm (Fig. 1A) revealed that TM-DS and TM-SO achieved maximal complex formation at TM-to-HIP agents ratios of 1.0:0.25 and 1.0:0.5, respectively. The zeta-potential of the TM alone was slightly positive, while all TM-HIP complexes displayed high negative surface charges, increasing with higher concentrations of HIP agent (Fig. 1B). This reflected the anionic nature of DS, SC, and SO, which impart additional negative charges to the particle surface (Sutar et al., 2021; Choi and Park, 2000; Sun et al., 2011). Among the complexes, TM-DS exhibited the highest precipitation efficiency (57.4 ± 3.5%), followed by TM-SO (26.8 ± 2.2%) at the optimized ratios (Fig. 1C), whereas TM-SC showed negligible precipitation. These results indicate that DS is the most suitable HIP agent for TM complexation due to its superior solubility at low pH, high precipitation efficiency, and favorable surface charge. The results are consistent with previous studies demonstrating the successful application of DS in hydrophobic ion-pair systems (Griesser et al., 2017; Yeerong et al., 2025).

**Fig. 2:**
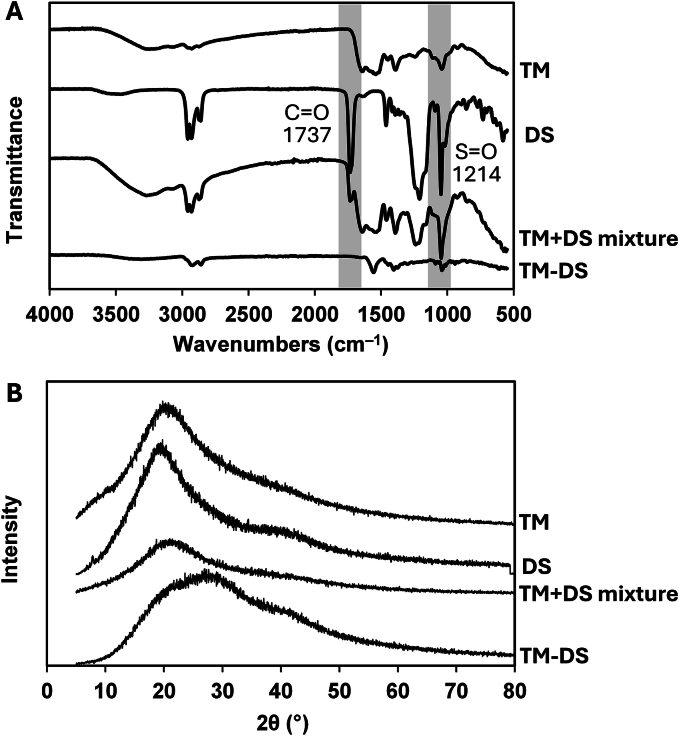
ATR-FTIR spectra (A) and XRD diffractograms (B) of *T. mitratus* cricket protein hydrolysate (TM), dioctyl sodium sulfosuccinate (DS), the physical mixture of TM and DS (TM + DS mixture), and the hydrophobic ion-pair complex of TM and DS (TM-DS).

The current study demonstrated that DS was the most suitable counterion due to its superior solubility at low pH and the highest precipitation efficiency. Optimal TM-DS complex formation was achieved at pH 2.0 with a TM-to-HIP agent mass ratio of 1:0.5, resulting in efficient precipitation and a favorable surface charge for subsequent formulation. Accordingly, the TM-HIP selected based on its highest precipitation efficiency (TM-DS) was further characterized using ATR-FTIR spectroscopy and XRD to confirm structural and functional interactions. The structural interactions between TM and DS, including both physical coexistence and chemical complexation, were analyzed, as shown in Fig. 2.

In the ATR-FTIR spectra (Fig. 2A), characteristic protein absorption bands were observed in TM, including the amide I (C=O stretch, ∼1650 cm^−1^) and amide II (N—H bending, ∼1540 cm^−1^) bands (Sadat and Joye, 2020; Haris and Severcan, 1999). DS exhibited distinct S=O stretching vibrations at 1214 cm^−1^ and C=O stretching at 1737 cm^−1^ (Sutar et al., 2021). In the physical mixture of TM and DS, both sets of peaks were present, indicating simple coexistence without strong interactions. In contrast, the TM-DS complex displayed noticeable shifts and intensity changes in the amide I and S=O regions, suggesting the formation of non-covalent interactions, such as electrostatic and hydrophobic interactions, between TM and DS (Sutar et al., 2021). These spectral changes confirm successful hydrophobic ion-pair complexation.

The XRD patterns (Fig. 2B) further supported these findings. TM and DS exhibited broad diffraction halos centered around 2*θ* ≈ 20°, indicating that both components are predominantly amorphous in nature. The physical mixture of TM and DS retained features of both components, indicating no significant structural alteration. In contrast, the TM-DS complex shows a noticeable change in the diffraction profile, including peak broadening and a shift in the maximum intensity position. These changes indicate a rearrangement of the molecular structure and a reduction in structural order compared to the physical mixture. The absence of sharp diffraction peaks further confirms that the TM-DS complex remains amorphous. The altered pattern suggested successful complex formation and intermolecular interactions between TM and DS. Therefore, the ATR-FTIR and XRD analyses demonstrated that the interaction of TM with DS leads to the formation of a stable TM-HIP complex through electrostatic and hydrophobic interactions, accompanied by changes in both functional group *v*ibrations and X-ray diffraction patterns. These structural modifications are expected to enhance the physicochemical stability and potential bioavailability of TM for subsequent nanocarrier formulations.

The lipophilicity of TM and the TM-DS complex was evaluated by determining the log D. In this study, *n*-butanol was used as the organic solvent, as TM was insoluble in *n*-octanol, the solvent typically used for log D measurements (Claus et al., 2023). TM exhibited a log D of −0.72 ± 0.03, indicating predominant hydrophilicity, whereas the TM-DS complex showed a log D of 0.38 ± 0.07, reflecting a shift toward increased lipophilicity. This change demonstrated that the formation of the TM-DS complex enhanced the hydrophobic character of the protein hydrolysate, which is expected to facilitate its encapsulation into lipid-based carriers and improve interaction with lipid membranes. The increase in lipophilicity is consistent with the principles of hydrophobic ion pairing (Claus et al., 2023), where the addition of appropriate counterions can mask charged groups and promote hydrophobic interactions, thereby improving partitioning into nonpolar phases.

### 3.3 Antioxidant activity of TM and TM-DS

**Fig. 3:**
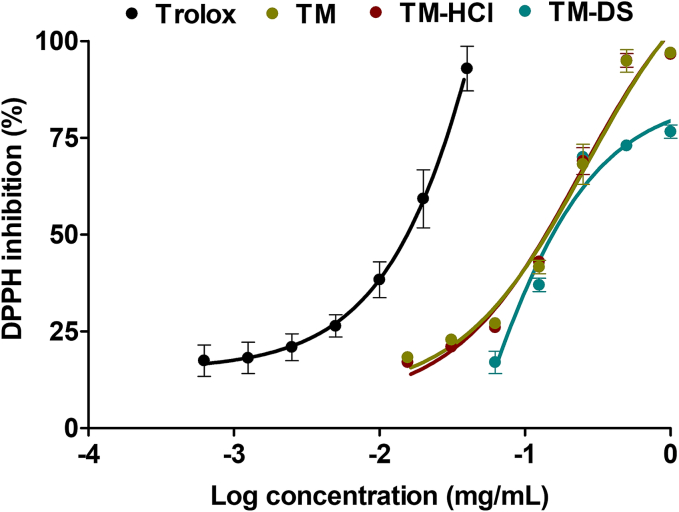
Dose–response curves of DPPH radical scavenging activity for Trolox, *T. mitratus* cricket protein hydrolysate aqueous solution (TM), *T. mitratus* cricket protein hydrolysate solution pH 2.0 (TM-HCl), and the complex of *T. mitratus* cricket protein hydrolysate and dioctyl sodium sulfosuccinate after dissociation (TM-DS).

The antioxidant activity of TM, evaluated through DPPH^•^ radical scavenging, exhibited a dose–response relationship, as shown in Fig. 3. In comparison, Trolox exhibited significantly greater antioxidant potency, as indicated by its lower IC_50_ value (33 ± 14 μg/ml) relative to TM (211 ± 33 μg/ml). Although TM was not directly comparable to the standard compound, it still exhibited appreciable antioxidant activity at higher concentrations, highlighting its potential as a natural antioxidant source. The IC_50_ value of 211 ± 33 μg/ml (≈0.021% *w*/*v*) suggested that the required concentration falls within a feasible range for practical applications. This antioxidant activity is likely attributed to the presence of bioactive peptides within the protein hydrolysate, whose activity depends on amino acid composition, sequence, and structure (Abeyrathne et al., 2022). These peptides can act as hydrogen or electron donors to neutralize free radicals, thereby mitigating oxidative damage (de Lima Cherubim et al., 2020). Antioxidants act by donating hydrogen atoms or electrons to neutralize free radicals, thereby interrupting oxidative chain reactions and reducing cellular damage, consequently, they help block the harmful effects of free radicals and support the normal production of structural proteins in the skin (de Lima Cherubim et al., 2020). These properties are particularly relevant for skin applications, as antioxidants help mitigate oxidative stress induced by environmental factors, prevent premature aging, and provide photoprotective and anti-inflammatory benefits (Arct and Pytkowska, 2008).

In the current study, the influence of acidic conditions associated with the HIP process was assessed. The IC_50_ value of TM in HCl solution at pH 2.0 (196 ± 29 μg/ml) was comparable to that of TM, suggesting that exposure to acidic conditions did not significantly impair its antioxidant activity. This suggested that the peptide structures responsible for radical scavenging are stable under acidic conditions, and that protonation does not significantly impair their functional activity. Furthermore, following HIP complexation and subsequent dissociation, TM-DS exhibited a comparable IC_50_value (192 ± 43 μg/ml), suggesting that the antioxidant functionality of the protein hydrolysate was largely preserved after the complexation and dissociation process. This finding indicated that the interactions involved in hydrophobic ion pairing are predominantly non-covalent and reversible, allowing the active peptide fractions responsible for radical scavenging to be recovered without significant degradation. The preservation of activity implies that key functional groups, such as amino, carboxyl, and hydrophobic residues involved in electron or hydrogen donation, remain structurally intact after processing. These findings indicate that TM exhibits promising antioxidant potential, supporting its applicability in skin-related formulations, while the HIP process allowed for its modification and recovery without compromising its functional bioactivity.

### 3.4 Nanocarriers containing TM

TM and TM-DS were successfully loaded into the various nanocarriers, all of which appeared as uniform, yellowish, translucent liquids (Fig. S5). Centrifugation at 5000 rpm for 15 min revealed slight sedimentation in the TM-CNP-NE, whereas the other formulations remained stable (Fig. S5A). Furthermore, all nanocarriers retained their original color and showed no signs of phase separation after six cycles of heating and cooling, indicating good physical stability under accelerated thermal stress (Fig. S5B). These observations suggested that the formulated nanocarriers can maintain structural integrity and visual homogeneity during handling and thermal fluctuations, which is critical for effective protein delivery applications.

The freshly prepared nanocarriers exhibited particle sizes ranged from ∼70–300 nm with narrow distributions (PDI 0.16–0.26), as shown in Table S1. TM-CNP was positively charged (+25 to +29 mV) due to chitosan, while TM-NE, TM-NLC, TM-CNP-NE, TM-DS-NE, and TM-DS-NLC were negatively charged (−22 to −42 mV) likely resulting from the anionic HIP complex and surfactants, indicating good colloidal stability (Table S1) (Danaei et al., 2018). These physicochemical properties suggested that all formulations possess good colloidal stability, which is expected to support effective protein delivery.

After six cycles of heating–cooling, most nanocarriers showed slight increases in particle size while maintaining narrow size distributions and acceptable surface charges. TM-CNP was the only formulation to exhibit a decrease in particle size, consistent with previous observations for chitosan nanoparticles *(**Inthorn et al.,* 2024*).* TM-CNP-NE displayed the largest size increase, likely due to partial neutralization of its surface charge resulting from interactions between negatively charged peptides or free amino acids in cricket protein and the positively charged chitosan. In contrast, lipid-based nanocarriers containing TM and TM-DS (both nanoemulsion and NLC) maintained nanoscale particle sizes, suggesting their potential suitability for enhanced skin penetration and stable protein delivery. Additionally, the lipid-based nanocarriers exhibited similar loading capacities, whereas TM-CNP showed the highest loading capacity (Table S2). This difference is likely due to the smaller internal phase of TM-CNP compared to the lipid-based systems, which increases the relative proportion of protein per unit mass of carrier.

**Fig. 4:**
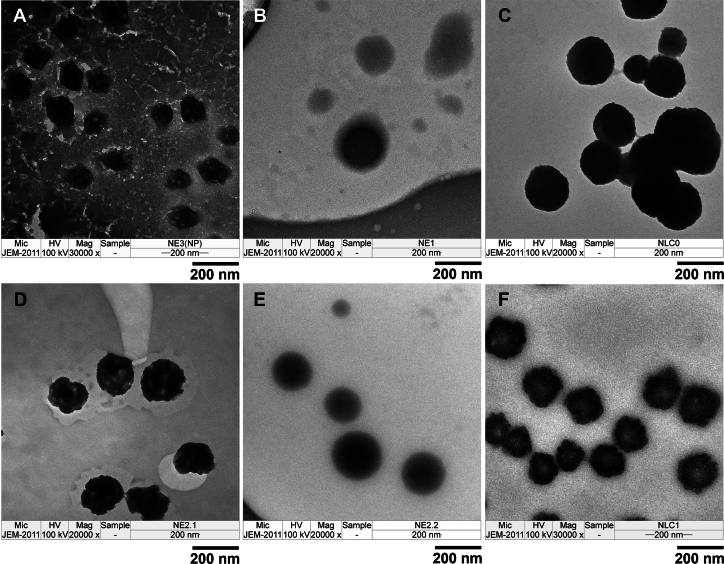
TEM images of chitosan nanoparticles with *T. mitratus* cricket protein hydrolysate (TM-CNP) (A), nanoemulsion containing the *T. mitratus* cricket protein hydrolysate (TM-NE) (B), nanostructured lipid carriers containing the *T. mitratus* cricket protein hydrolysate (TM-NLC) (C), nanoemulsion containing chitosan nanoparticles with *T. mitratus* cricket protein hydrolysate (TM-CNP-NE) (D), nanoemulsion containing the complex of *T. mitratus* cricket protein hydrolysate and dioctyl sodium sulfosuccinate (TM-DS-NE) (E), and nanostructured lipid carriers containing the complex of *T. mitratus* cricket protein hydrolysate and dioctyl sodium sulfosuccinate (TM-DS-NLC) (F).

TEM images (Fig. 4) confirmed discrete, non-aggregated particles for all nanocarriers. TM-CNP and TM-CNP-NE exhibited rough surface morphologies without signs of aggregation, which is consistent with previous reports (Tang et al., 2003; Phan, 2021). In contrast, lipid-based nanocarriers containing TM and TM-DS (both nanoemulsion and NLC) displayed fine droplet structures. TM-NE and TM-DS-NE exhibited a round and smooth morphology, with no irregular particulates observed. TM-NLC and TM-DS-NLC, the semi-solid lipid formulations, showed a comparatively irregular surface morphology relative to nanoemulsion, which may be attributed to partial recrystallization of the solid lipid matrix within the NLC. The particle sizes observed by TEM were in good agreement with those determined by dynamic light scattering.

### 3.5 EE and LC of nanocarriers containing TM

**Fig. 5:**
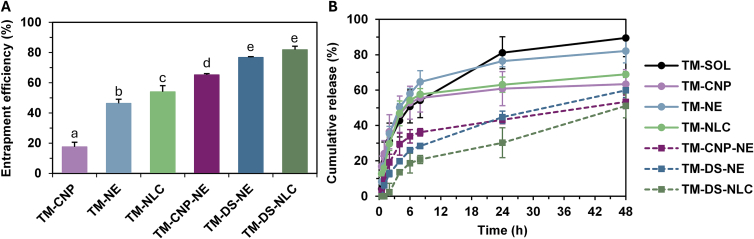
Entrapment efficiency (A) and release profiles (B) of *T. mitratus* cricket protein hydrolysate over 48 h for *T. mitratus* cricket protein hydrolysate aqueous solution (TM-SOL), chitosan nanoparticles with *T. mitratus* cricket protein hydrolysate (TM-CNP), nanoemulsion containing the *T. mitratus* cricket protein hydrolysate (TM-NE), nanostructured lipid carriers containing the *T. mitratus* cricket protein hydrolysate (TM-NLC), nanoemulsion containing chitosan nanoparticles with *T. mitratus* cricket protein hydrolysate (TM-CNP-NE), nanoemulsion containing the complex of *T. mitratus* cricket protein hydrolysate and dioctyl sodium sulfosuccinate (TM-DS-NE), and nanostructured lipid carriers containing the complex of *T. mitratus* cricket protein hydrolysate and dioctyl sodium sulfosuccinate (TM-DS-NLC). Lowercase letters (a–e) indicate significant differences determined by one-way ANOVA followed by Tukey's post hoc test (*p* < 0.05) using GraphPad Prism version 10.0.

EE of TM varied significantly among the nanocarriers as shown in Fig. 5A. TM-CNP showed the lowest entrapment (17.6 ± 3.1%), whereas lipid-based systems demonstrated substantially higher encapsulation, with TM-NE and TM-NLC achieving moderate EE values (46.0 ± 2.8% and 54.0 ± 4.0%, respectively). A further increase was observed upon incorporation of HIP, where TM-CNP-NE, TM-DS-NE, and TM-DS-NLC exhibited progressively higher efficiencies, reaching 65.2 ± 0.8%, 76.8 ± 0.5%, and 81.9 ± 2.4%, respectively. These differences likely reflect the effects of carrier composition and HIP-induced charge neutralization of TM, which enhances lipophilicity and protein encapsulation within nanocarriers. The low EE observed for TM-CNP can be attributed to the high water solubility of TM. As the protein readily dissolves in the aqueous phase of the nanocarrier system, a smaller fraction remains entrapped within the chitosan polymer matrix, resulting in reduced encapsulation efficiency. In contrast, lipid-based carriers (nanoemulsion and NLC) improved EE due to their ability to accommodate TM within the dispersed lipid phase. Notably, direct comparisons within the same carrier systems clearly demonstrate the contribution of HIP. EE increased from 46.0 ± 2.8% (TM-NE) to 65.2 ± 0.8% (TM-CNP-NE) and further to 76.8 ± 0.5% (TM-DS-NE). The likely explanation is that complexation as TM-CNP or TM-DS improved EE, with CNP stabilizing the protein to reduce leakage and DS neutralizing charges to enhance its association with the carrier lipids. Similarly, for the NLC systems, EE rose from 54.0 ± 3.2% (TM-NLC) to 81.9 ± 2.4% (TM-DS-NLC). The highest EE values observed in both TM-DS-NE and TM-DS-NLC confirm that TM-DS forms a complex with lipophilic characteristics, which promotes its retention within the lipid droplets of the lipid-based nanocarriers for both nanoemulsion and NLC. These findings are consistent with earlier reports demonstrating that NLC can efficiently encapsulate peptide–hydrophobic ion-pair complexes (Liu et al., 2025). Moreover, the results align with previous studies showing that HIP can enhance the EE of peptides in nanoemulsion from 48% to 88% (Jeon et al., 2025). The LC values of the nanocarrier formulations are presented in Table S2. All nanocarriers demonstrated promising protein loading, with TM-CNP showing the highest LC of 1.5 ± 0.3%, while the others were between 0.3% to 0.4%. This result is consistent with previous studies, in which the loading capacity of tripeptides incorporated into nanocarriers was reported to be approximately 0.4–0.6% (Dymek et al., 2025). Moreover, some studies have reported that even at a low concentration of 0.1% *w/w*, macromolecular proteins such as hyaluronic acid were able to penetrate the skin *in vitro* when incorporated into phospholipid-based nanocarriers (Chen et al., 2014). Notably, all formulations contained the same final amount of cricket protein to ensure consistency in subsequent membrane penetration studies.

### 3.6 *In vitro* release of TM from nanocarriers

The release profiles of TM from the aqueous solution (TM-SOL) and from the nanocarriers are shown in Fig. 5B. TM-SOL, TM-CNP, TM-NE, and TM-NLC exhibited rapid release, with approximately 50% released within the first 6 h and up to 60% by 24 h. In contrast, TM-SOL alone reached a cumulative release of 89.5 ± 10.6% over the same period, while TM-NE reached 82.1 ± 6.7%. TM-CNP-NE, TM-DS-NE and TM-DS-NLC showed a slower initial release of approximately 20% within 6 h, followed by sustained release reaching ∼50% over 48 h. The faster release from TM-SOL compared to chitosan nanoparticles is consistent with previous reports (Yeerong et al., 2024a). Moreover, TM-DS-NLC exhibited significantly slower release than TM-CNP formulations, in agreement with earlier studies showing sustained release from lipid-based nanocarriers (Li et al., 2016; Akel et al., 2021).

Within the same lipid systems, TM-CNP-NE and TM-DS-NE showed slower and more controlled release (53.3 ± 6.8% and 59.9 ± 3.9%, respectively) compared to TM-NE (82.1 ± 6.7%) at 48 h. A similar trend was observed for NLCs, with TM-DS-NLC releasing less (51.1 ± 4.0%) than TM-NLC (68.9 ± 1.7%) at the same period. These comparisons within identical carriers indicate that the differences are primarily due to the presence of the complexes rather than the lipid matrix. CNP likely stabilized the protein via electrostatic and steric effects, reducing leakage, while DS reduced residual charges, enhancing lipid association and controlling release. These findings support the conclusion that HIP complexation modulated release behavior by reducing the apparent aqueous solubility of TM and increasing its affinity for the lipid phase, thereby limiting diffusion into the external medium. While the lipid matrix provides an additional diffusional barrier, direct comparisons confirm that the sustained release effect is significantly enhanced by both CNP and the ion-pairing strategy with DS.

### 3.7 Strat-M® membrane permeation and retention

**Fig. 6:**
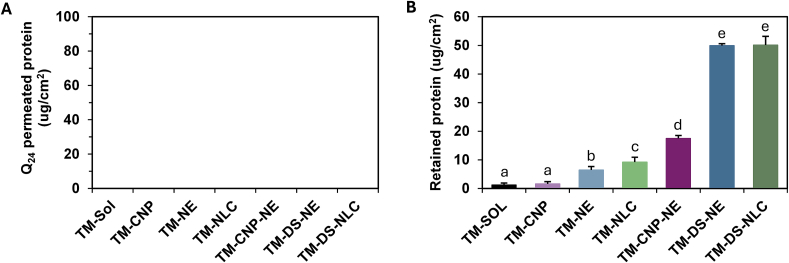
Membrane permeation (A) and retention (B) of *T. mitratus* cricket protein hydrolysate over 24 h for *T. mitratus* cricket protein hydrolysate aqueous solution (TM-SOL), chitosan nanoparticles with *T. mitratus* cricket protein hydrolysate (TM-CNP), nanoemulsion containing the *T. mitratus* cricket protein hydrolysate (TM-NE), nanostructured lipid carriers containing the *T. mitratus* cricket protein hydrolysate (TM-NLC), nanoemulsion containing chitosan nanoparticles with *T. mitratus* cricket protein hydrolysate (TM-CNP-NE), nanoemulsion containing the complex of *T. mitratus* cricket protein hydrolysate and dioctyl sodium sulfosuccinate (TM-DS-NE), and nanostructured lipid carriers containing the complex of *T. mitratus* cricket protein hydrolysate and dioctyl sodium sulfosuccinate (TM-DS-NLC). No permeation was detectable in panel (A) for all formulations under the experimental conditions. Lowercase letters (a–e) indicate significant differences determined by one-way ANOVA followed by Tukey's post hoc test (p < 0.05) using GraphPad Prism version 10.0.

Strat-M® membranes were used to evaluate the permeation and retention of nanocarriers containing TM and TM-DS. As an artificial membrane lacking protein components, Strat-M® minimizes analytical interference and is thus suitable for this assessment. The permeation and retention profiles of protein from different formulations are shown in Fig. 6. No detectable protein permeation into the receiver medium was observed for any formulation over 24 h (Fig. 6A), indicating limited membrane permeability. Consequently, the protein content in the receiver medium could not be measured, suggesting that topical application of these nanocarriers is unlikely to result in systemic exposure or related side effects (Chaiyana et al., 2020).

In contrast, measurable protein amounts were retained within the Strat-M® membrane for all formulations after 24 h (Fig. 6B). TM-SOL (1.2 ± 0.7 μg/cm^2^) and TM-CNP (1.6 ± 0.8 μg/cm^2^) exhibited minimal retention. Lipid-based nanocarriers significantly enhanced retention within the Strat-M® membrane for both nanoemulsion and NLC systems, exhibiting retention values of 6.4 ± 1.2 μg/cm^2^ and 9.2 ± 1.7 μg/cm^2^, respectively (*p* < 0.05). Additionally, TM-CNP-NE demonstrated significantly higher retention (17.5 ± 1.1 μg/cm^2^) compared to TM-CNP, suggesting the potential of lipid-based nanocarriers to enhance Strat-M® retention of TM. The highest protein delivery into the membrane was observed for TM-DS-NE (49.9 ± 0.7 μg/cm^2^) and TM-DS-NLC (50.1 ± 3.1 μg/cm^2^). These results indicate that lipid-based nanocarriers containing TM-DS significantly enhance protein retention within the membrane compared to chitosan-based systems.

TM-CNP did not enhance membrane retention compared with TM-SOL. The limited retention observed for both TM-SOL and TM-CNP is likely due to the hydrophilic nature of TM and the chitosan-based carrier, which is poorly compatible with the hydrophobic Strat-M® membrane that models the outer layers of human skin (Mortazavi and Moghimi, 2022). In contrast, TM-CNP-NE achieved significantly higher retention within the Strat-M® membrane than TM-SOL and TM-CNP. This increase could be attributed to the lipid-based composition of the nanoemulsion in TM-CNP-NE compared with TM-CNP alone or TM-SOL, which promotes improved membrane affinity and penetration compared with TM-CNP and TM-SOL (Magrode et al., 2024). Moreover, protein membrane retention from TM-DS-NE was significantly higher than that from TM-CNP-NE, indicating that the enhanced lipophilicity conferred by TM-DS improves membrane retention relative to hydrophilic TM and chitosan-based formulations.

TM-DS-NE and TM-DS-NLC exhibited comparable levels of protein membrane retention, both of which were significantly higher than those observed for the other formulations. These results indicate that retention within the Strat-M® membrane is enhanced by the combination of lipid-based nanocarriers and TM-DS, which increases the lipophilicity of the protein and promotes its interaction with the lipophilic membrane. Therefore, the findings suggest that lipid-based nanocarriers (both nanoemulsions and NLCs) may be effective delivery systems for improving the dermal retention of TM-DS. Their small particle size, compatibility with skin lipids, and high entrapment efficiency likely contribute to the enhanced protein deposition within the membrane (Adib et al., 2016; Venkatesan et al., 2025). However, it should be noted that the Strat-M® membrane is a simplified synthetic model that does not fully replicate the complex structure, enzymatic activity, and metabolic environment of human skin. Thus, the present results should be interpreted as indicative of relative formulation performance rather than definitive of in vivo dermal delivery. Further evaluation using ex vivo human or porcine skin would be suggested to confirm the translational relevance of these findings.

## 4 Conclusion

This study demonstrated that HIP effectively improved the compatibility of TM with lipid-based nanocarriers, enhancing encapsulation and retention within the Strat-M® membrane. TM successfully formed a HIP complex with DS, with the DS concentration strongly influencing complex formation. Log D measurements confirmed that the TM-DS exhibited increased lipophilicity (log D = 0.38 ± 0.07) compared to TM alone (log D = −0.72 ± 0.03). CNPs, nanoemulsions, and NLC formulations incorporating TM or TM-DS were successfully prepared, exhibiting nanoscale particle sizes, narrow size distributions, and stable zeta potential. Lipid-based nanocarriers containing TM-DS showed the highest encapsulation efficiency and sustained release, while chitosan nanoparticles demonstrated lower protein loading. TM-DS-NE and TM-DS-NLC achieved the greatest membrane retention, with NLC identified as the most effective carrier overall. These findings highlight the synergistic effects of protein lipophilicity, carrier composition, and nanoscale size in enhancing topical protein delivery, providing a promising approach to develop safe and efficient protein-based therapeutics. Future studies should evaluate these formulations in ex vivo human skin or clinical models to confirm their efficacy and safety under physiologically relevant conditions.

## CRediT authorship contribution statement

**Jirasit Inthorn:** Writing – review & editing, Writing – original draft, Visualization, Methodology, Investigation, Funding acquisition, Formal analysis. **Suvimol Somwongin:** Validation, Methodology, Funding acquisition. **Saranya Juntrapirom:** Validation, Methodology. **Watchara Kanjanakawinkul:** Resources, Methodology, Conceptualization. **Andrea Heinz:** Writing – review & editing, Writing – original draft, Validation, Supervision, Funding acquisition. **Anette Müllertz:** Writing – review & editing, Writing – original draft, Validation, Supervision. **Thomas Rades:** Writing – review & editing, Writing – original draft, Validation, Supervision, Methodology, Conceptualization. **Wantida Chaiyana:** Writing – review & editing, Writing – original draft, Validation, Supervision, Resources, Project administration, Methodology, Funding acquisition, Conceptualization.

## Declaration of competing interest

The authors declare that they have no known competing financial interests or personal relationships that could have appeared to influence the work reported in this paper.

## Data Availability

Data will be made available on request.
