# Supplementary material for: Enhanced encapsulation and membrane retention of Teleogryllus mitratus protein via hydrophobic ion-pairing in nanostructured lipid carriers
Source: Int J Pharm X. 2026 Apr 24;11:100550. doi: 10.1016/j.ijpx.2026.100550 (PMC13141765; doi:10.1016/j.ijpx.2026.100550)
Supplement: Supplementary file 1 — Supplementary material [file mmc1.docx]

***Supplementary material***

**Enhanced encapsulation and membrane retention of *Teleogryllus mitratus* protein via hydrophobic ion-pairing in nanostructured lipid carriers**

Jirasit Inthorn^a^, Suvimol Somwongin^a^, Saranya Juntrapirom^b^, Watchara Kanjanakawinkul^b^, Andrea Heinz^c,e^, Anette Müllertz^d,e^, Thomas Rades^e^, Wantida Chaiyana^a,f,g,h,^*

^a^ Department of Pharmaceutical Sciences, Faculty of Pharmacy, Chiang Mai University, Chiang Mai 50200, Thailand

^b^ Chulabhorn Royal Pharmaceutical Manufacturing Facilities by Chulabhorn Royal Academy, Chon Buri 20180, Thailand

^c^ Department of Pharmacy, LEO Foundation Center for Cutaneous Drug Delivery, University of Copenhagen, 2100, Copenhagen, Denmark

^d^ Bioneer: FARMA, Department of Pharmacy, University of Copenhagen, Universitetsparken 4, Copenhagen, 2100, Denmark

^e^ Department of Pharmacy, Faculty of Health and Medical Sciences, University of Copenhagen, Universitetsparken 2, 2100, Copenhagen, Denmark

^f^ Center of Excellence in Pharmaceutical Nanotechnology, Faculty of Pharmacy, Chiang Mai University, Chiang Mai 50200, Thailand

^g^ Research Center of Deep Technology in Beekeeping and Bee Products for Sustainable Development Goals (SMART BEE SDGs), Chiang Mai University, Chiang Mai 50200, Thailand

^h^ Multidisciplinary and Interdisciplinary School, Chiang Mai University, Chiang Mai 50200, Thailand

*Corresponding author.

Tel.: +66‒53‒944343

E-mail: wantida.chaiyana@cmu.ac.th (W. Chaiyana)

**Table S1**. Particle size, polydispersity index (PDI), and zeta potential of nanocarriers containing TM, before and after heat–cool storage for six cycles

| **Nanocarriers** | **Particles size (nm)** | | **PDI** | | **Zeta potential (mV)** | |
| --- | --- | --- | --- | --- | --- | --- |
|  | **Before** | **After** | **Before** | **After** | **Before** | **After** |
| TM-CNP | 298 ± 20 | 286 ± 0^*^ | 0.23 ± 0.01 | 0.24 ± 0.00 | 25 ± 3 | 29 ± 1^*^ |
| TM-NE | 74 ± 1 | 185 ± 6^*^ | 0.24 ± 0.01 | 0.28 ± 0.01^*^ | −37 ± 6 | −29 ± 6^*^ |
| TM-NLC | 83 ± 3 | 198 ± 1^*^ | 0.16 ± 0.01 | 0.25 ± 0.01^*^ | −39 ± 1 | −36 ± 3^*^ |
| TM-CNP-NE | 70 ± 4 | 252 ± 6^*^ | 0.26 ± 0.01 | 0.18 ± 0.01^*^ | −22 ± 1 | −42 ± 1^*^ |
| TM-DS-NE | 91 ± 1 | 165 ± 1^*^ | 0.21 ± 0.01 | 0.12 ± 0.02^*^ | −34 ± 3 | −35 ± 0^*^ |
| TM-DS-NLC | 100 ± 1 | 176 ± 1^*^ | 0.17 ± 0.05 | 0.13 ± 0.03^*^ | −30 ± 2 | −37 ± 1^*^ |

Data are expressed as mean ± SD, *n* = 3. ^*^ indicates a statistically significant difference between before and after heat–cool storage for six cycles, as determined by a paired *t*-test (*p* < 0.05).

**Table S2**. Loading capacity of nanocarriers containing TM.

| **Nanocarriers** | **Loading capacity (% w/w)** |
| --- | --- |
| TM-CNP | 1.5 ± 0.3 ^b^ |
| TM-NE | 0.2 ± 0.0 ^a^ |
| TM-NLC | 0.3 ± 0.0 ^a^ |
| TM-CNP-NE | 0.3 ± 0.0 ^a^ |
| TM-DS-NE | 0.3 ± 0.0 ^a^ |
| TM-DS-NLC | 0.4 ± 0.0 ^a^ |

Data are expressed as mean ± SD, n = 3. Different lowercase letters (a, b) indicate statistically significant differences among formulations, determined by one-way ANOVA followed by Tukey’s post hoc test (p < 0.05).

**
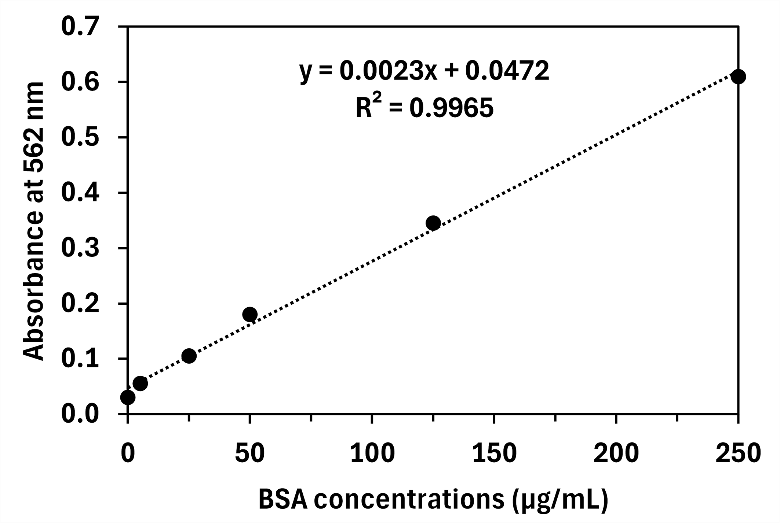
**

**Fig. S1.** Calibration curve of bovine serum albumin (BSA) in the bicinchoninic acid (BCA) assay (0–250 µg/mL). The linear regression equation (y = 0.0023x + 0.0472, R² = 0.9965) was used to determine protein content.


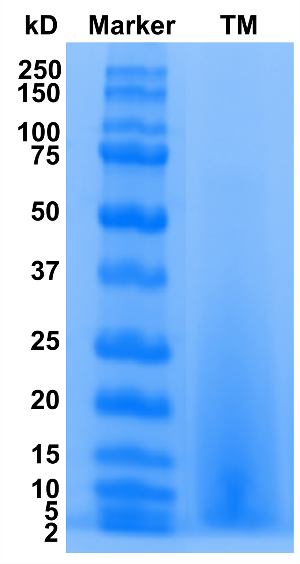


**Fig. S2.** SDS–PAGE protein profile of *T. mitratus* cricket protein hydrolysate (TM). The gel (12% polyacrylamide) was stained with Coomassie Brilliant Blue G-250. The left lane shows the molecular weight marker (2–250 kDa) and the right lane represents the TM.

**
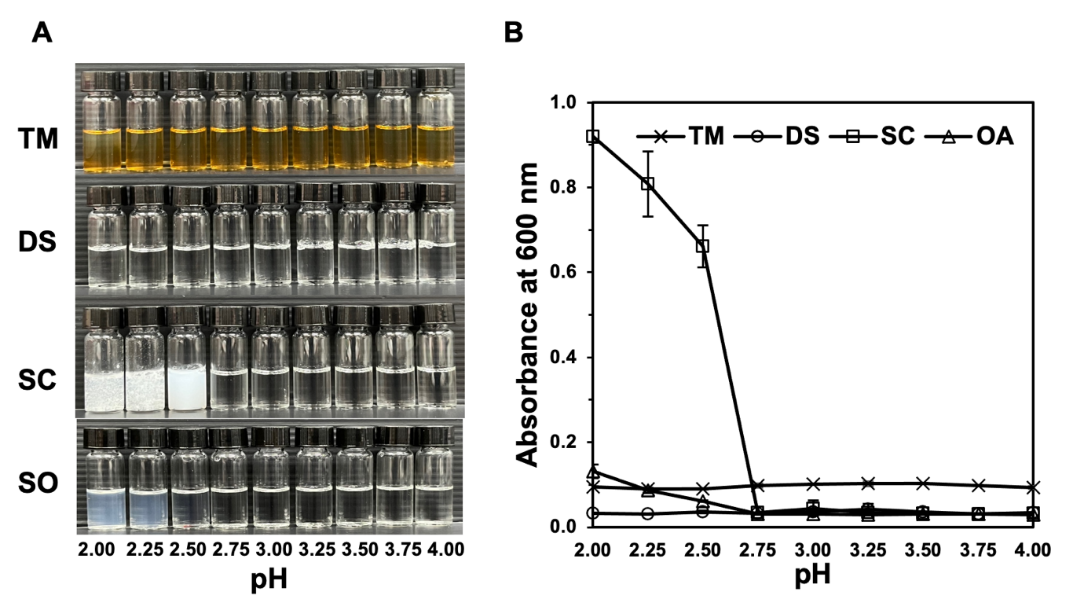
**

**Fig. S3.** External appearance (A) and turbidity (B) of *T. mitratus* cricket protein hydrolysate (TM), dioctyl sodium sulfosuccinate (DS), sodium deoxycholate (SC), and sodium oleate (SO) aqueous solutions at different pH levels.


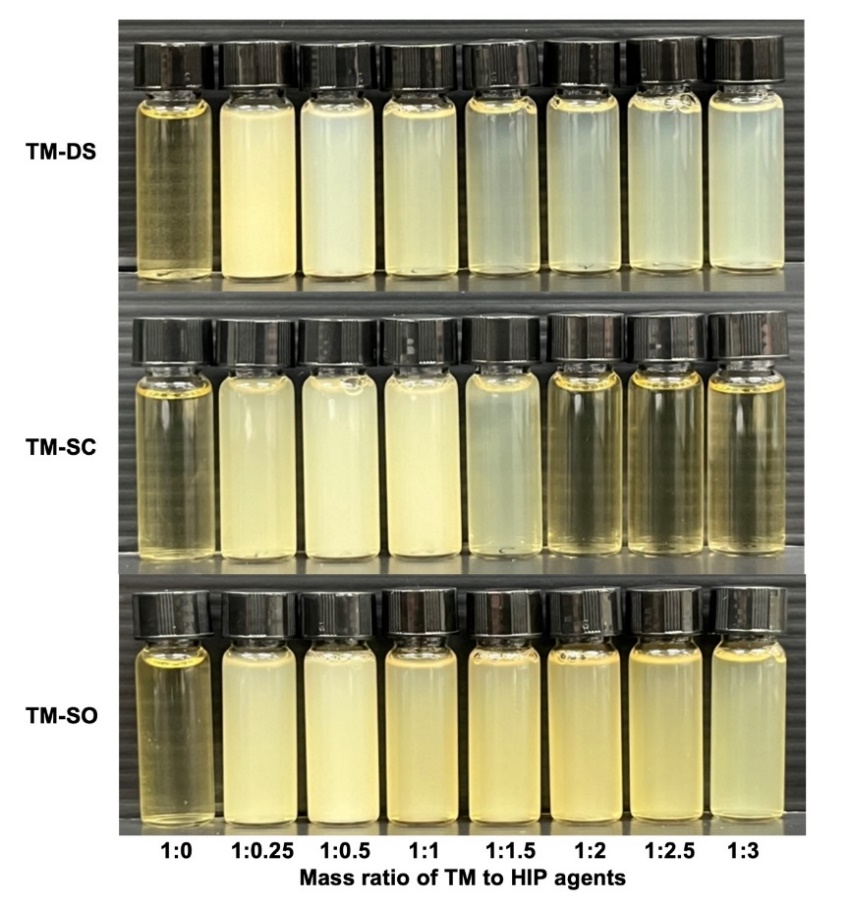


**Fig. S4.** External appearance of TM-HIP prepared at different mass ratios using various hydrophobic ion-pairing (HIP) agents, including dioctyl sodium sulfosuccinate (TM-DS), sodium deoxycholate (TM-SC), and sodium oleate (TM-SO).

**
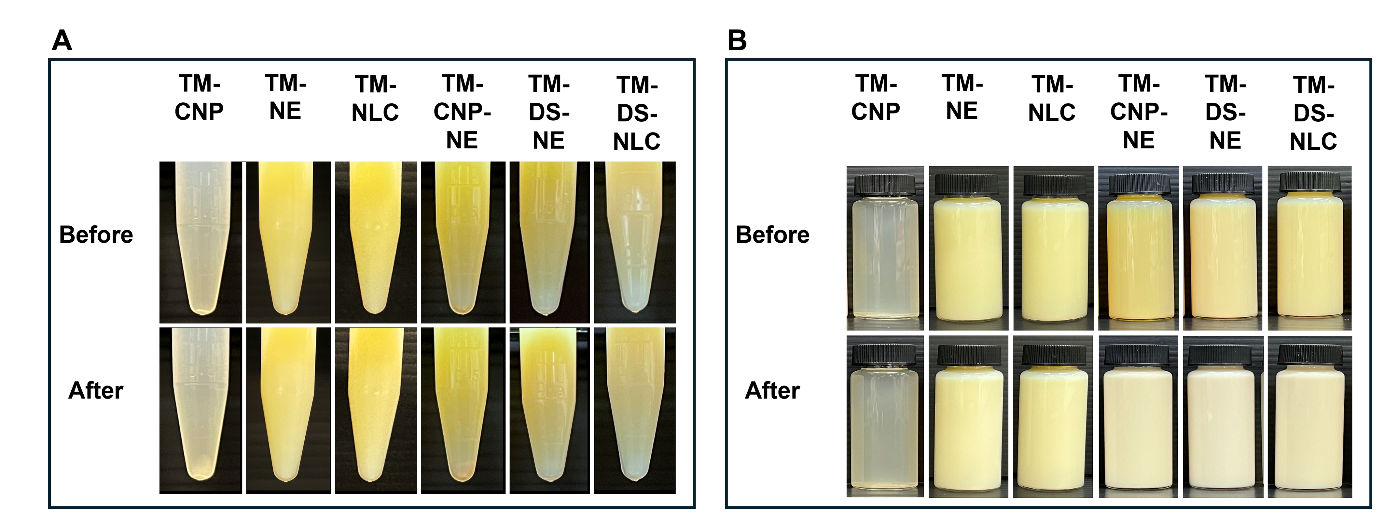
**

**Fig. S5.** External appearance of nanocarrier formulations before and after centrifugation (A) and under six heating–cooling cycles (B) for chitosan nanoparticles with *T. mitratus* cricket protein hydrolysate (TM-CNP), nanoemulsion containing the *T. mitratus* cricket protein hydrolysate (TM-NE), nanostructured lipid carriers containing the *T. mitratus* cricket protein hydrolysate (TM-NLC), nanoemulsion containing chitosan nanoparticles with *T. mitratus* cricket protein hydrolysate (TM-CNP-NE), nanoemulsion containing the complex of *T. mitratus* cricket protein hydrolysate and dioctyl sodium sulfosuccinate (TM-DS-NE), and nanostructured lipid carriers containing the complex of *T. mitratus* cricket protein hydrolysate and dioctyl sodium sulfosuccinate (TM-DS-NLC).
